# Nationwide surveillance detects yellow fever and chikungunya viruses in multiple *Aedes* mosquito species in Nigeria

**DOI:** 10.1186/s13071-025-07051-z

**Published:** 2025-10-31

**Authors:** Udoka C. Nwangwu, Harouna Soumare, Judith U. Oguzie, William E. Nwachukwu, Cosmas O. Onwude, Festus A. Dogunro, Mawlouth Diallo, Chukwuebuka K. Ezihe, Nneka O. Agashi, Emelda I. Eloy, Stephen O. Anokwu, Clementina C. Anioke, Linda C. Ikechukwu, Chukwuebuka M. Nwosu, Oscar N. Nwaogo, Ifeoma M. Ngwu, Rose N. Onyeanusi, Angela I. Okoronkwo, Francis U. Orizu, Monica O. Etiki, Esther N. Onuora, Peter C. Okeke, Okechukwu C. Chukwuekezie, Josephine C. Ochu, Anise N. Happi, Sulaiman S. Ibrahim, Adetifa Ifedayo, Chikwe Ihekweazu, Christian T. Happi

**Affiliations:** 1 National Arbovirus and Vectors Research Centre (NAVRC), Enugu, Enugu State Nigeria; 2 African Centre of Excellence for Genomics of Infectious Diseases (ACEGID), Redeemer’s University, Ede, Osun State Nigeria; 3 Department of Biological Sciences, Faculty of Natural Sciences, Redeemer’s University, Ede, Osun State Nigeria; 4 Nigeria Centre for Disease Control, Abuja, FCT Nigeria; 5 Medical Zoology Center, Institut Pasteur de Dakar, Dakar, Senegal; 6 Malaria Consortium, Abuja, FCT Nigeria; 7 Department of Parasitology and Entomology, Nnamdi Azikiwe University, Awka, Anambra State Nigeria; 8 Opec Research Consult, Awka, Anambra State Nigeria; 9 Department of Biochemistry, Bayero University, Kano, Kano State Nigeria; 10 Centre for Research in Infectious Diseases (CRID), Yaoundé, Cameroon; 11 Department of Immunology and Infectious Diseases, Harvard T.H. Chan School of Public Health, Harvard University, Boston, MA USA

**Keywords:** Yellow fever, Chikungunya, Arboviruses, *Aedes* mosquitoes, Minimum infection rates, Vector control

## Abstract

**Background:**

Since the reemergence of yellow fever in 2017, the Nigeria Centre for Disease Control (NCDC) has been coordinating responses to local outbreaks with the support of the World Health Organization (WHO). The National Arbovirus and Vectors Research Centre (NAVRC) has been implementing targeted vector control interventions to mitigate the occurrence of these outbreaks. This study sought to identify the vectors driving yellow fever (YF) transmission and other public health arboviruses and their distribution across Nigeria.

**Methods:**

Between 2017 and 2020, larvae, pupae, and adult mosquitoes were collected, largely targeting *Aedes* Stegomyia mosquitoes, in observational, analytical, and cross-sectional surveys conducted in sixteen YF outbreak states of Nigeria. Adult mosquitoes (field-collected or reared from immature stages) were morphologically identified, and arboviruses were detected using reverse transcription quantitative polymerase chain reaction (RT-qPCR).

**Results:**

Seven different *Aedes* mosquito species were found in 11 of the 16 states surveyed, with mosquitoes from nine states found infected with arboviruses. *Aedes aegypti* was the predominant species (51%), whereas *Aedes africanus* was the least common species (0.2%). Yellow fever virus (YFV) was detected in 33 (~ 26%) out of the 127 *Aedes* mosquito pools, with minimum infection rates in the ranges of 0.9 (*Ae. circumluteolus*) to 62.5 (*Ae. luteocephalus*) per 1000 mosquitoes. In addition to YFV, the chikungunya virus (CHIKV) was found in nine pools, with minimum infection rates ranging from 1.6 (*Ae. aegypti*) to 62.5 (*Ae. luteocephalus*) per 1000 mosquitoes. Except for *Ae. africanus*, all the *Aedes* species tested positive for at least one arbovirus. YFV-positive pools were found in six *Aedes* species (*Ae. aegypti*, *Ae. albopictus*, *Ae. simpsoni* complex, *Ae. luteocephalus*, *Ae. vittatus*, and *Ae. circumluteolus*), while CHIKV-positive pools were recorded in only two *Aedes* species (*Ae. aegypti* and *Ae. luteocephalus*). There was co-detection of YFV and CHIKV in *Ae. luteocephalus* (Benue State). Edo State had the most positive pools (16), while Nasarawa, Imo, and Anambra states had the least (one positive pool). Breteau and house indices were higher than the standard WHO transmission thresholds in all but one state, suggesting high risk for arbovirus transmission.

**Conclusions:**

In Nigeria, there is substantial risk of arbovirus transmission by *Aedes* mosquitoes, with YFV posing a large threat. This risk is heightened by the fact that YFV and CHIKV have been detected concurrently in vectors across outbreak locations. There is an urgent need to step up arbovirus surveillance and vector control activities across the country.

**Graphical Abstract:**

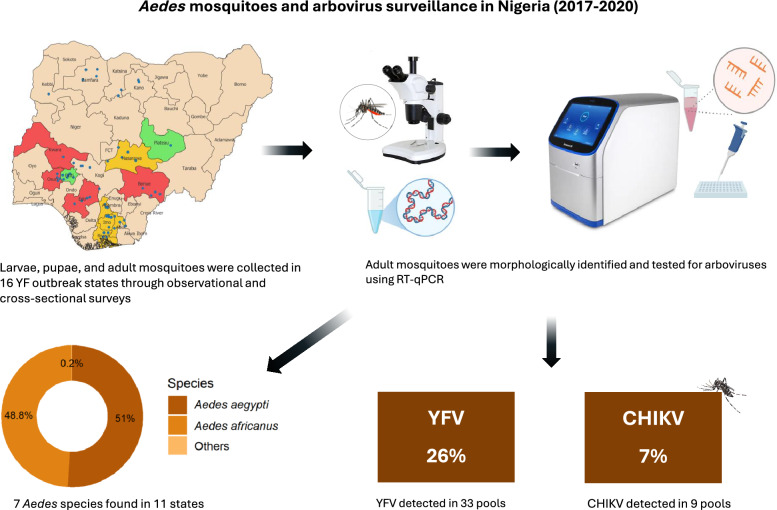

**Supplementary Information:**

The online version contains supplementary material available at 10.1186/s13071-025-07051-z.

## Background

The encroachment of invasive *Aedes* mosquitoes is increasing the spread of debilitating arboviral infections, such as dengue, chikungunya, and yellow fever viruses, among others [1]. These *Aedes* species have spread to all continents, except Antarctica [2], where they transmit major arboviruses of public health importance. Globalization, the increasing volume and pace of trade and travel, continuing urbanization, and environmental challenges, which include climate change, are the major factors driving the spread of these vectors and the diseases they transmit [3].

*Aedes aegypti* and *Ae. albopictus* are the two most medically important *Ae.* species [4, 5]. Together, they are largely responsible for the transmission of dengue, chikungunya, yellow fever, and Zika viruses around the world [4, 6]. However, *Ae.* *albopictus* has been implicated in the transmission of these diseases in temperate regions [7, 8] as well as tropical Africa [9–11]. While dengue, chikungunya, yellow fever, and Zika viruses all have sylvatic cycles involving forest mosquitoes and non-human primates, recent global outbreaks have been dominated by urban transmission via *Ae*. *aegypti* and *Ae*. *albopictus* [4]. In African settings, *Ae. aegypti* and *Ae. albopictus* are also considered to be the major arbovirus vectors [12]. However, several minor *Aedes* mosquitoes are epidemiologically significant secondary vectors of arboviruses. These include *Aedes africanus*, *Ae. luteocephalus*, *Ae. simpsoni* complex, *Ae. vittatus*, *Ae. metallicus*, *Ae. opok*, and the *Ae. furcifer*/*taylori* group [13]. Most of these species are found in Nigeria [14], and some have been found to harbor yellow fever and dengue viruses in different parts of the country [15, 16]. The distribution of these vectors is mostly rural or sylvatic [15, 16]. Sylvatic dengue viruses in Africa are transmitted among non-human primates by *Ae. furcifer* and *Ae. luteocephalus* and usually cross over to humans through biting by *Ae. furcifer* [17].

Funding gaps hugely affect the control of arboviruses in Africa. According to the WHO [18], most countries reported a lack of adequate financial and technical support. The WHO also highlighted that 34 countries (72%) in the WHO African region reported that they did not have an emergency fund or a specified emergency funding mechanism for arbovirus disease outbreak response in the previous two years. Consequently, there is a lack of training and retraining for staff involved in the surveillance and control of arboviral diseases, and a lack of community awareness of arboviral diseases, as well as systematic surveillance and reporting system [19]. Diagnostic capacity for dengue, as for virtually all causes of acute febrile illness (AFI), is limited in Africa. It is essential to understand the biology and behavior of local vectors because these factors will influence transmission, as well as the selection and design of effective control tools and strategies [20]. Unfortunately, data regarding vectors and disease transmission is insufficient in most parts of Africa [12]. The spread of arboviruses (chikungunya, dengue, and Zika viruses) in Africa is, to a large extent, not properly understood. Also, knowledge of the differences in the risk of transmitting these diseases and yellow fever within the subregions is inhibited by a lack of data [21, 22].

In Africa, yellow fever outbreaks have predominantly been reported in rural or sylvatic contexts, where transmission involves spillover from non-human primates and sylvatic *Aedes* vectors into human populations [13]. This epidemiological pattern highlights the importance of rural communities in sustaining outbreaks. Since the earliest recorded outbreak of yellow fever in Nigeria in 1864 [23], the disease has been a recurrent public health challenge in the country [24]. However, there have been quiescent periods and resurgences. Yellow fever outbreaks have overlapped with outbreaks or cases of other arboviruses, which are often not reported or underreported, as they have usually had a smaller impact on morbidity and mortality [25–27]. Moreover, the similarity of signs and symptoms as well as the poor diagnosis have been a major challenge to delineating these arboviruses for proper mapping in the country. This is also a limitation to measuring the impact of the individual disease burden. There have been several reports of other arboviruses, including Zika virus disease [28, 29], dengue [25, 27], and chikungunya [25, 26] in several parts of Nigeria. Unfortunately, there are usually no follow-ups and measurable impacts, and these viruses may have remained unnoticed in circulation for ages.

The resurgence of yellow fever in Nigeria started in Ifelodun Local Government Area (LGA) of Kwara State in 2017 [30]. Since 2017, yellow fever has been reported in all 36 states and Federal Capital Territory (FCT), with 32 states reporting at least one confirmed case [31]. Between September 2017 and December 2021, there were a total of 14,272 suspected cases from 759 (98.0%) LGAs across all states in Nigeria. Of these 14,272 suspected cases, 702 were confirmed by reference laboratories.

Hence, in line with the national yellow fever outbreak response strategy, we carried out a nationwide entomological surveillance to investigate the arboviruses in circulation and the *Aedes* species that transmit them across Nigeria. Here we provide information on vector distribution and composition, which will guide the relevant authorities to formulate policies that will bring about the prevention and control of these vectors and the arboviral diseases they transmit.

## Methods

### Study area

**Fig. 1:**
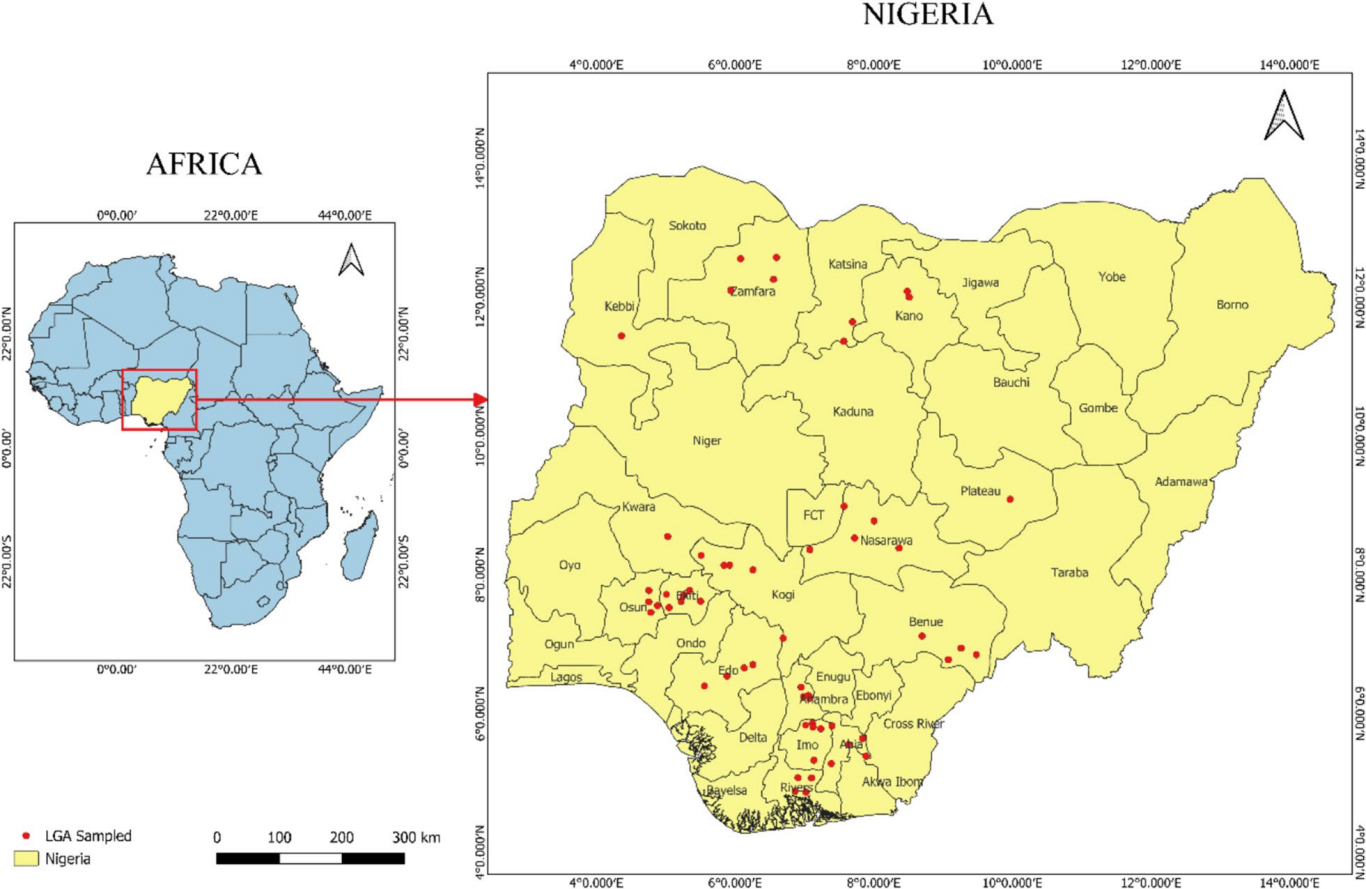
Map of Nigeria showing local government areas sampled in different states

The study was conducted in 57 local government areas (LGAs) across 16 states in Nigeria (Fig. 1). Most of the areas are rural settlements where farming is the primary occupation. Nigeria is characterized by high temperatures reaching up to 32 °C in the coastal south and up to 41 °C in the north [32] from six ecological zones, spanning from south to north: mangrove swamp, freshwater swamp, rainforest, Guinea savanna, Sudan savanna, and Sahel savanna [33]. The climate varies from very wet, typical in coastal areas in the south of Nigeria, with an annual rainfall greater than 3500 mm, to dry in the Sahel region in the northwest and northeast parts, with annual rainfall below 600 mm per annum, and huge climatic variations depending on the regions, with the climate becoming drier along a latitudinal gradient from south to north. There are two seasons—rainy (April to October) and dry (November to March), characterized by the harmattan, especially in the north. The harmattan season begins in November/December and ends in February.

### Mosquito collection

The survey used entomological approaches, including larval surveys, modified human landing catch (mHLC), BG-Sentinel trap, and CDC light trap, largely targeting *Aedes* (*Stegomyia*) species. These were deployed in various locations across seasons (rainy and dry) depending on the time of the outbreak. Each visit/response to a YF outbreak lasted 8–10 days.

#### Larval survey

Using the house of a yellow fever case as the center, larval sampling was carried out in houses within a 300-m radius. This activity targeted the immature stages of domestic and peridomestic breeders among the *Aedes* species [13]. Artificial containers in and around various houses were sampled for larvae and pupae of the vectors. Plant axils (natural habitats) that might harbor immature stages (larvae and/or pupae) of *Aedes* mosquitoes around human dwellings were also sampled. Where possible, the entire water content of a container was emptied into a bowl and the immature stages were picked using pipettes. Otherwise, the container was recorded as positive and a household member assisted by using a cup or small container to scoop any larvae and/or pupae present from the water surface of the larger container. The larvae and pupae were transferred into labeled containers and transported to the field base for rearing. After field activities, they were moved to the centre’s insectary, where rearing was completed to the adult stage for morphological identification and further analyses.

#### Modified human landing catch (mHLC)

This method was carried out outdoors following a well-established protocol [13], though modified by covering most parts of the body. Two individuals performed this across outbreak locations. Adult collections were performed in the mornings between 07:00 and 10:00 am and then evenings between 4 and 8 pm in the same location, over 2–3 days. Biting activities of most *Aedes* species peaked during these periods [34–36]. Mosquitoes were caught using mouth aspirators or glass test tubes as soon as they landed to bite. Those collected with mouth aspirators were transferred to borosilicate test tubes. The test tubes were quickly plugged with cotton wool and transferred to cold boxes with ice packs.

All participants involved in the modified human landing catches (mHLC) provided informed consent after being fully briefed on the objectives, procedures, potential risks, and their right to withdraw at any stage. Collectors were trained and protective measures were applied to minimize risk.

#### Adult collection using BG-Sentinel trap (Biogents Sentinel trap)

Adult *Aedes* mosquitoes were collected using BG-Sentinel traps (Biogents, Regensburg, Germany) baited with BG-Lure^®^. This trap targets day-blood-seeking female mosquitoes [13]. At each study location, two traps were deployed per sampling day, placed at least 20–30 m apart to avoid trap interference. Traps were positioned outdoors in shaded, cool areas of the peridomestic environment, with some near vegetation and others closer to houses to capture variation in resting/host-seeking behavior. Collections were carried out for 12 h daily (07:00–19:00 h), after which the traps were retrieved and mosquitoes were collected using aspirators and transferred into well-labeled paper cups covered with untreated nets. Each site was sampled for the number of days the visit lasted (2–3 days). Sampling was carried out both in the rainy and dry seasons, depending on the time of the outbreak.

#### Adult collection using CDC light trap

To investigate the nocturnal and crepuscular activity of *Aedes* species (particularly the sylvatic species), two CDC light traps were deployed per site. However, owing to site-specific constraints, trap deployment varied and was not conducted at all sites. Traps were positioned outdoors (one per house), approximately 5–10 m in front of human dwellings and suspended 1.5 m above the ground. Where possible, one trap was placed under or close to tall trees, and the other closer to household surroundings, to capture variation in mosquito resting and host-seeking behavior. This placement style was alternated every day. Each trapping session lasted from 18:00 to 06:00 h. At the end of each trapping period (3 h), the collection cup was retrieved and replaced and the contents examined for adult mosquitoes. Mosquitoes were collected using aspirators and transferred into well-labeled paper cups covered with untreated nets. Sampling was carried out for two to three consecutive nights per site.

### Morphological identification and preservation of mosquitoes

Adult mosquitoes collected in the field and those reared to adults from the immature stages were chilled to death or knocked down using ethyl acetate. There in the field, the specimens were morphologically identified on chill tables or ice packs, using the taxonomic keys [37–39]. They were then introduced into well-labeled Eppendorf tubes containing RNAlater. As soon as the team returned from the field, the tubes were eventually stored at –20 °C in the laboratory.

### Identification of arboviruses using RT-qPCR

Mosquitoes were pooled according to species, sex, and location. Each pool contained an average of 23 mosquitoes (Table 3). Mosquito pools were homogenized in 1 mL of cooled Dulbecco’s modified Eagle’s medium (DMEM) (composition of 500 ml DMEM high glucose (4.5 g/L) with l-glutamine), 1 mL penicillin–streptomycin, 15 mL fetal calf serum (FCS) 3%, and 5 mL amphotericin B) and 500 ml of zirconia beads (2.0 mm, cat. no 1107912; Firma Biospec). The contents were macerated using the Qiagen Tissuelyser LT for 10 min and then centrifuged at 4500 × *g* for 15 min. Viral RNA was extracted from supernatant using the Qiagen viral QIAamp mini kit (Qiagen, City, Country), and RT-qPCR was used to screen flaviviruses (YFV, WNV, DENV, and ZIKV) and alphaviruses (CHIK and ONNV).

SYBR green RT-qPCR was performed on a Roche LightCycler 96. Samples were run in triplicate and considered positive only if amplification was observed in all replicates, with nuclease-free water included as non-template control (NTC). Each reaction contained 3 μL of RNA template and 7 μL of reaction mixture, consisting of 1.32 μL nuclease-free water, 5 μL Power SYBR Green Master Mix, 0.08 μL of 125× reaction mix, and 0.3 μL each of forward and reverse primers at a final concentration of 0.3 μM.

Real-time RT-qPCR amplification was carried out for 45 cycles at 48 °C for 30 min, 95 °C for 10 min, 95 °C for 15 s, and 60 °C for 30 s. Temperatures for the melt curves were 95 °C for 15 s, 55 °C for 15 s, and 95 °C for 15 s for all previously published primers used in this study [40–44]. We used a cycle threshold (Ct) cutoff of 40 to define positive results by RT-qPCR. All samples were run in triplicate, with both positive and negative controls included. A list of these primers is provided in Table 1.

**Table 1:** Primer sequences for genotyping arboviruses

Genus-level/species-specific assay	Forward and reverse primers
Pan-flavivirus	TACAACATGATGGGAAAGAGAGAGAARAA GTGTCCCAKCCRGCTGTGTCATC
Pan-alphavirus	YAGAGCDTTTTCGCAYSTRGCHW CATRAANKGNGTNGTRTCRAANCCDAYCC
YFV	GCTAATTGAGGTGYATTGGTCTGC CTGCTAATCGCTCAAMGAACG
WNV	GGGCCTTCTGGTCGTGTTC GATCTTGGCYGTCCACCTC
ZIKV	AARTACACATACCARAACAAAGTG GT TCCRCTCCCYCTYTGGTCTTG
CHIKV	GACAATGCGCGCGGTACC TGTTGTTTTGTGGCGCCT
ONNV	CAGTGATCCCGAACACGGTG CCACATAAATGGGTAGACGCC
Pan-dengue	TTGAGTAAACYRTGCTGCCTGTAGCTC GAGACAGCAGGATCTCTGGTCTYTC

### Data analyses

Mosquito count data were analyzed using a generalized linear mixed-effects model (GLMM) with a negative binomial distribution to account for overdispersion. Fixed effects included mosquito species, collection method, and their interaction, while state was incorporated as a random intercept to account for location-level variation. Model selection supported the final model (Akaike information criterion = 679.0; Bayesian information criterion = 755.2; log likelihood = −316.5), and a dispersion parameter of 0.315 confirmed model adequacy.

For the larval survey, the Breteau index (BI; the number of positive containers per 100 houses), container index (CI; the percentage of water-holding containers that were positive for larvae and/or pupae), and house index (HI; the percentage of houses with at least one positive container) were estimated. Arbovirus transmission risk was classified using WHO criteria with high risk as BI > 50, HI > 35, or CI > 20; moderate risk as BI between 5 and 50, indicating vector densities sufficient to sustain an outbreak; and low risk as BI < 5, HI < 4, and CI < 3 [45]. Any location with an active outbreak and the presence of an established arbovirus vector was also classified as high risk [46].

Preferred breeding sites (PBS) of *Aedes* species were determined by calculating the percentage of containers with water (PCW) and the percentage of positive containers (PPC) for each container type. PBS was expressed as the ratio of PPC to PCW, with higher values indicating greater breeding preference.

Minimum infection rates (MIR), defined as the number of infected mosquitoes per 1000 tested, were estimated using the PooledInfRate package. A Poisson generalized linear model (GLM) was then applied to assess the relationship between MIR for yellow fever and chikungunya viruses and factors such as the number of mosquitoes per pool and the number of pools tested.

All statistical analyses were conducted in R (version 4.5.0) and Python. GLMMs were fit using the glmmTMB package, post hoc comparisons and visualizations were performed with emmeans, and MIR estimation was carried out using the pooledBin function from the PooledInfRate package. Statistical significance was set at *P* < 0.05. Data are presented in bar charts, distribution maps, and tables.

## Results

### Composition, richness, and regional distribution of *Aedes* species

**Fig. 2:**
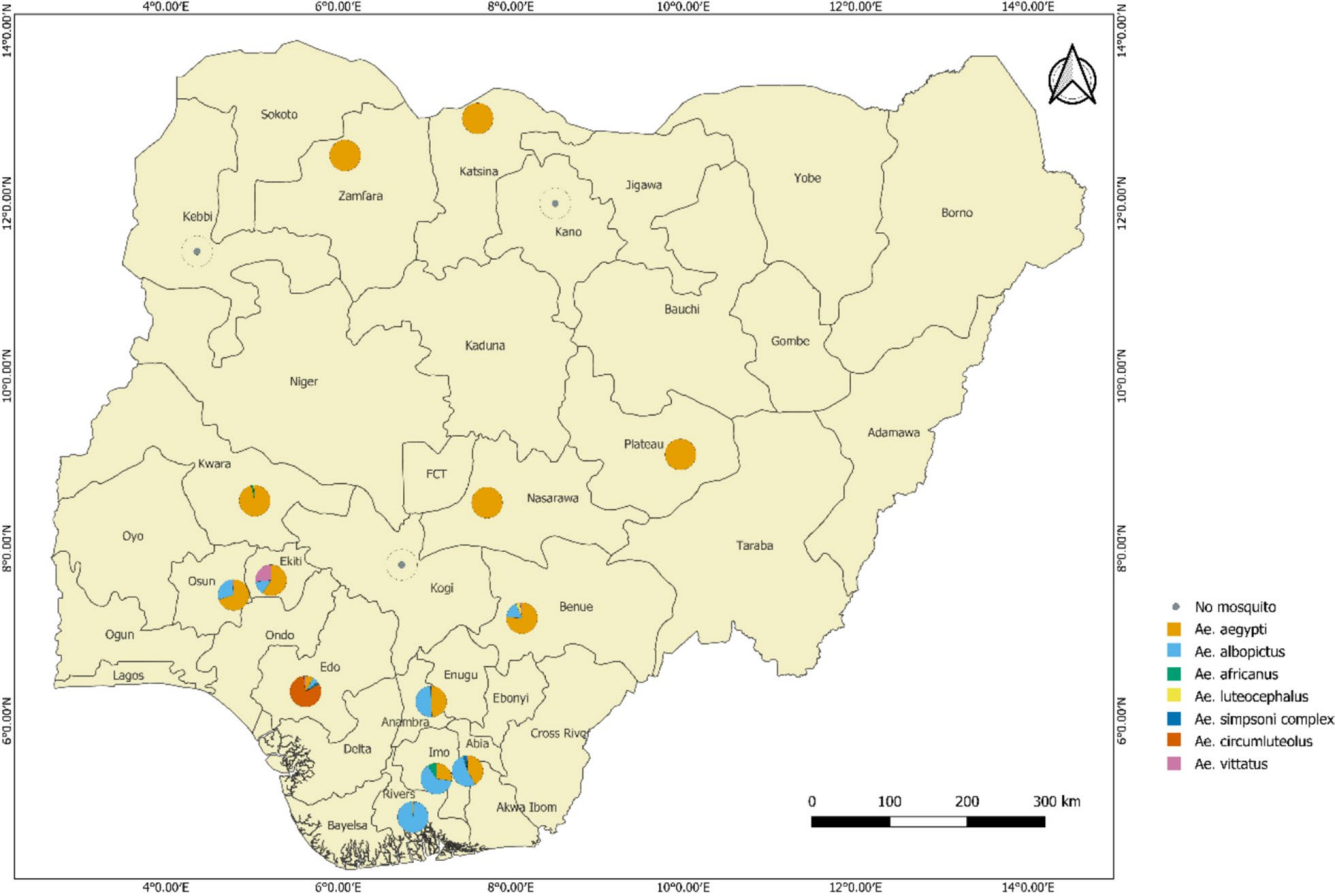
Species distribution across the states where mosquitoes were sampled

A total of 2406 *Aedes* mosquitoes, representing seven species, were collected across 16 states during yellow fever outbreak investigations (Fig. 2). Five species belonged to the subgenus *Stegomyia* (*Ae. aegypti*, *Ae. albopictus*, *Ae. simpsoni* complex, *Ae. luteocephalus*, and *Ae. africanus*), one to *Aedimorphus* (*Ae. vittatus*), and one to *Neomelaniconion* (*Ae. circumluteolus*). Species richness increased gradually from the drier northern zones to the wetter southern regions.

In the South West and South South zones, Edo State showed the highest richness, with six of the seven species recorded in the study, while *Ae. circumluteolus* dominated collections, albeit largely restricted to a single community. *Ae. aegypti* was most abundant in Osun State, while *Ae. albopictus* predominated in Rivers State. In the South East zone, *Ae. albopictus* was overwhelmingly dominant, followed by *Ae. aegypti*. In the North West zone, only *Ae. aegypti* was reported from Katsina and Zamfara states, while none of the mosquitoes were collected in Kano and Kebbi states. The North Central zone yielded five species, including *Ae. aegypti*, *Ae. albopictus*, *Ae. africanus*, *Ae. vittatus*, and *Ae. luteocephalus*. Individual counts of species by state are presented in Additional file 7: Table S4.

**Fig. 3:**
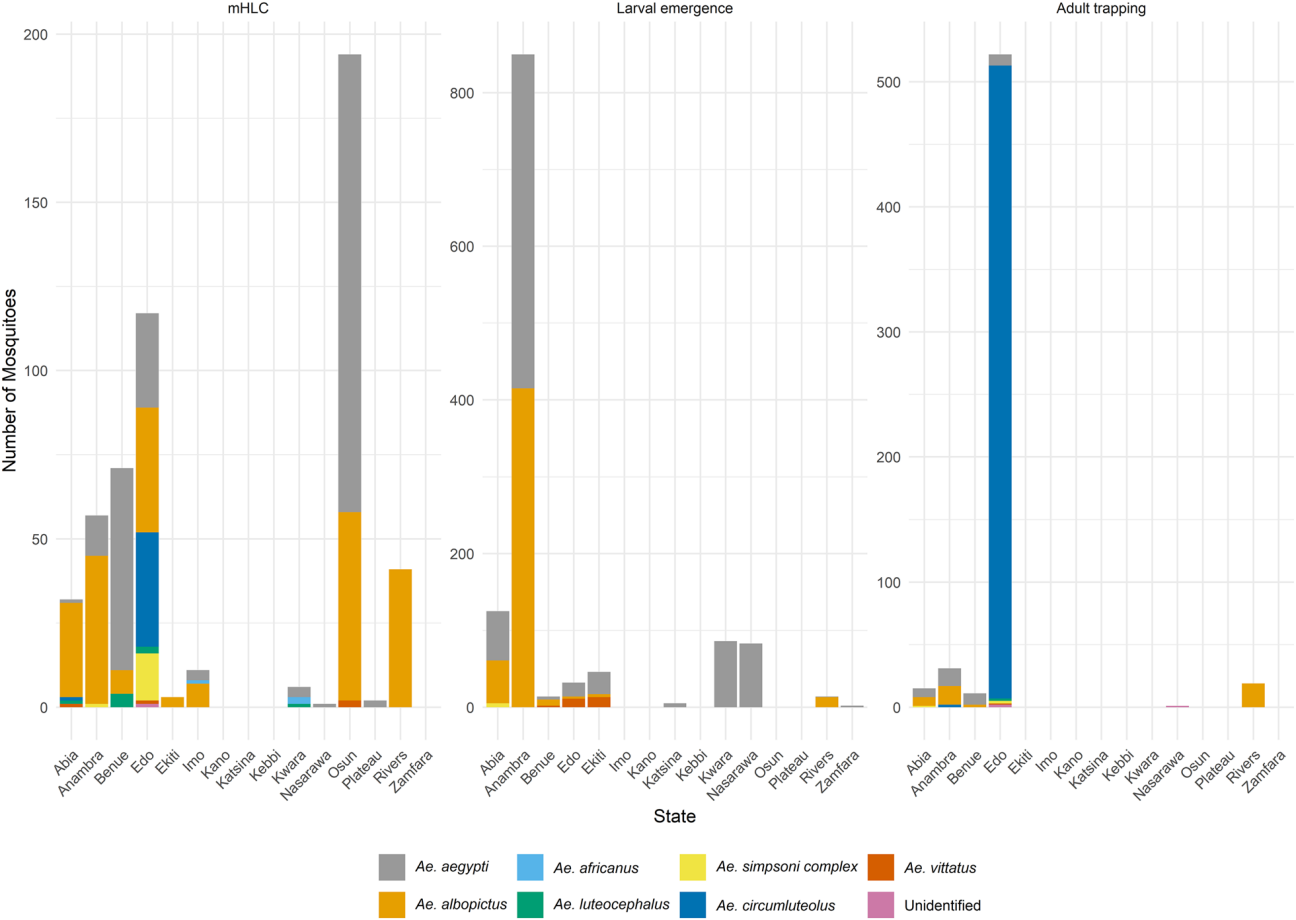
Mosquito species by state and collection method

Analysis of estimated marginal means from the negative binomial mixed-effects model revealed clear differences in mosquito abundance across species and sampling methods (Fig. 3). Results were back-transformed from the log scale, with 95% confidence intervals applied. Across all methods, *Ae. aegypti* and *Ae. albopictus* were the most abundant species, while other species such as *Ae. africanus*, *Ae. circumluteolus*, *Ae. luteocephalus*, *Ae. simpsoni complex*, and *Ae. vittatus* generally exhibited lower estimated counts.

For the mHLC method, *Aedes aegypti* had a mean abundance of 6.98 (95% confidence interval (CoI) [1.25, 39.00]), while *Ae. albopictus* showed a mean of 7.30 (95% CoI [1.32, 40.00]). Other species had substantially lower estimates, all below 1. Pairwise contrasts revealed that *Ae. aegypti* was significantly more abundant than *Ae. africanus*, *Ae. circumluteolus*, *Ae. luteocephalus*, *Ae. simpsoni* complex, and *Ae. vittatus* (all *P*-values < 0.05). However, no significant difference was found between *Ae. aegypti* and *Ae. albopictus* (*P* = 1.00), indicating comparable dominance between these species in HLC collections.

For the larval collection method, *Ae. aegypti* had a mean abundance of 49.81 (95% CoI [9.73, 255.00]), while *Ae. albopictus* had a mean of 10.66 (95% CoI [1.98, 57.00]). Several species, including *Ae. africanus*, *Ae. circumluteolus*, and *Ae. luteocephalus*, were not found in larval samples. Pairwise contrasts revealed that *Ae. aegypti* was significantly more abundant than *Ae. simpsoni* complex (*P* < 0.001) and *Ae. vittatus* (*P* = 0.0045), while *Ae. albopictus* was also significantly more abundant than *Ae. simpsoni* complex (*P* = 0.0017). No other contrasts were statistically significant.

For the adult trapping method, abundance was more evenly distributed, with *Ae. albopictus* showing a mean abundance of 3.80 (95% CoI [0.47, 31.00]), *Ae. aegypti* showing 1.57 (95% CoI [0.21, 12.00]), and *Ae. circumluteolus* showing 2.69 (95% CoI [0.27, 26.00]). Significant differences were observed between *Ae. albopictus* and *Ae. luteocephalus* (*P* = 0.0276) and between *Ae. albopictus* and *Ae. vittatus* (*P* = 0.0303). Similarly, *Ae. circumluteolus* was significantly more abundant than *Ae. luteocephalus* (*P* = 0.0244) and *Ae. vittatus* (*P* = 0.0302).

### Epidemic risk indices and breeding preferences across states

**Fig. 4:**
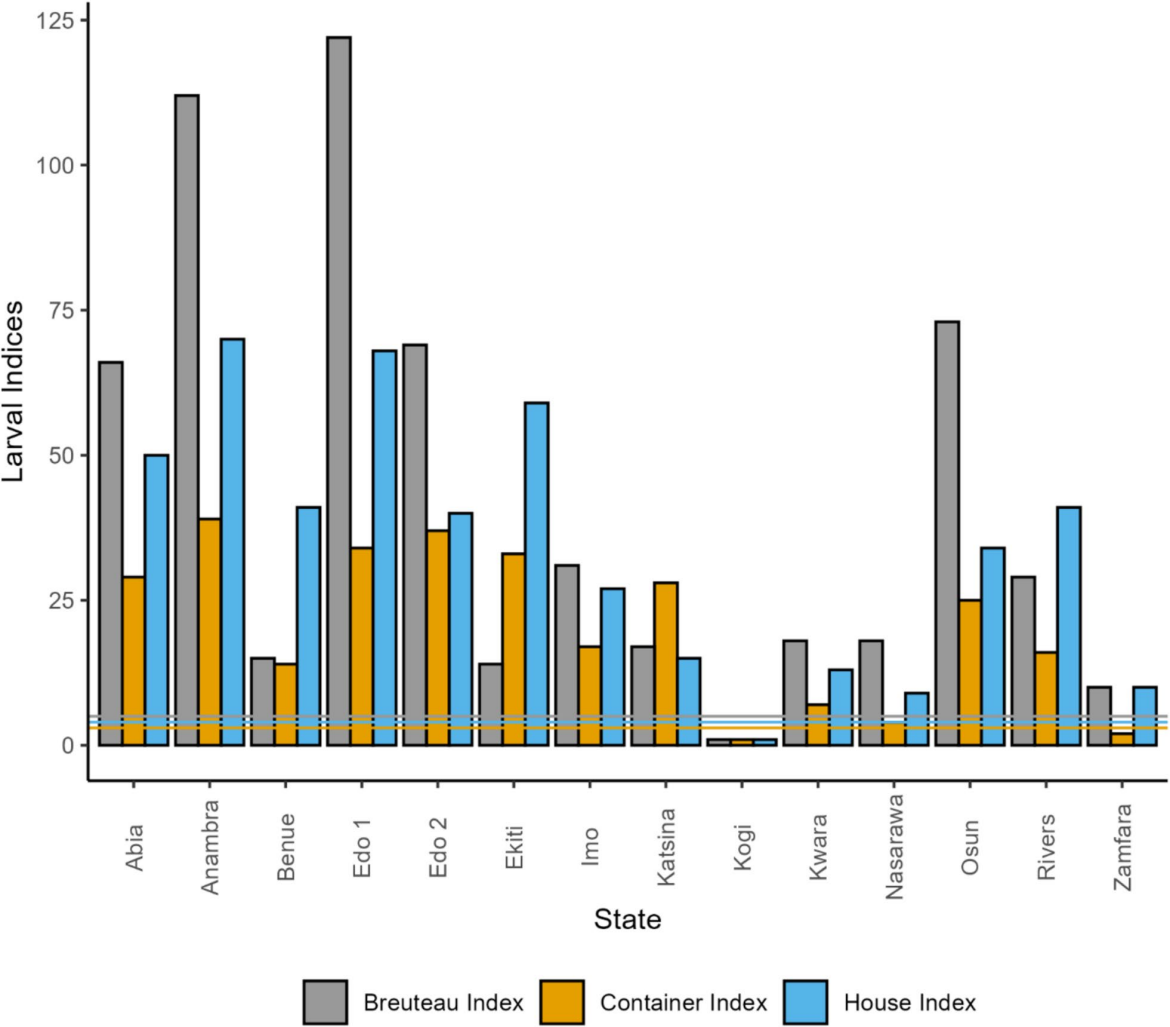
Epidemic risk indices across sampled states. The thresholds for the Breteau, container, and house indices are 5%, 3%, and 4% according to the WHO (threshold lines are indicated in the figure)

Results of the epidemic risk indices show that the house and Breteau indices were higher, respectively, than the 4% and 5% thresholds set by the WHO for epidemic risk across the states where larval surveys were successful, except for Kogi. The container index was also higher than this standard threshold (3%) in all localities except Kogi and Zamfara states (Fig. 4).

The container typology results suggest that the preferred breeding sites of *Aedes* mosquitoes in the study areas are tyres, earthen pots/wares, discarded containers, other water storage containers, and leaf/plant axils (Additional file 1: Fig. S1). Earthen pots and tyres seems the preferred breeding sites in northern Nigeria (Additional file 2: Fig. S2). In contrast, the vectors preferred tyres as well as discarded and other materials in the southern parts of the country (Additional file 3: Fig. S3). However, mosquitoes collected from Rivers and Ekiti states showed a striking preference for tyres.

### Mosquito infectivity with arboviruses across the states

**Fig. 5:**
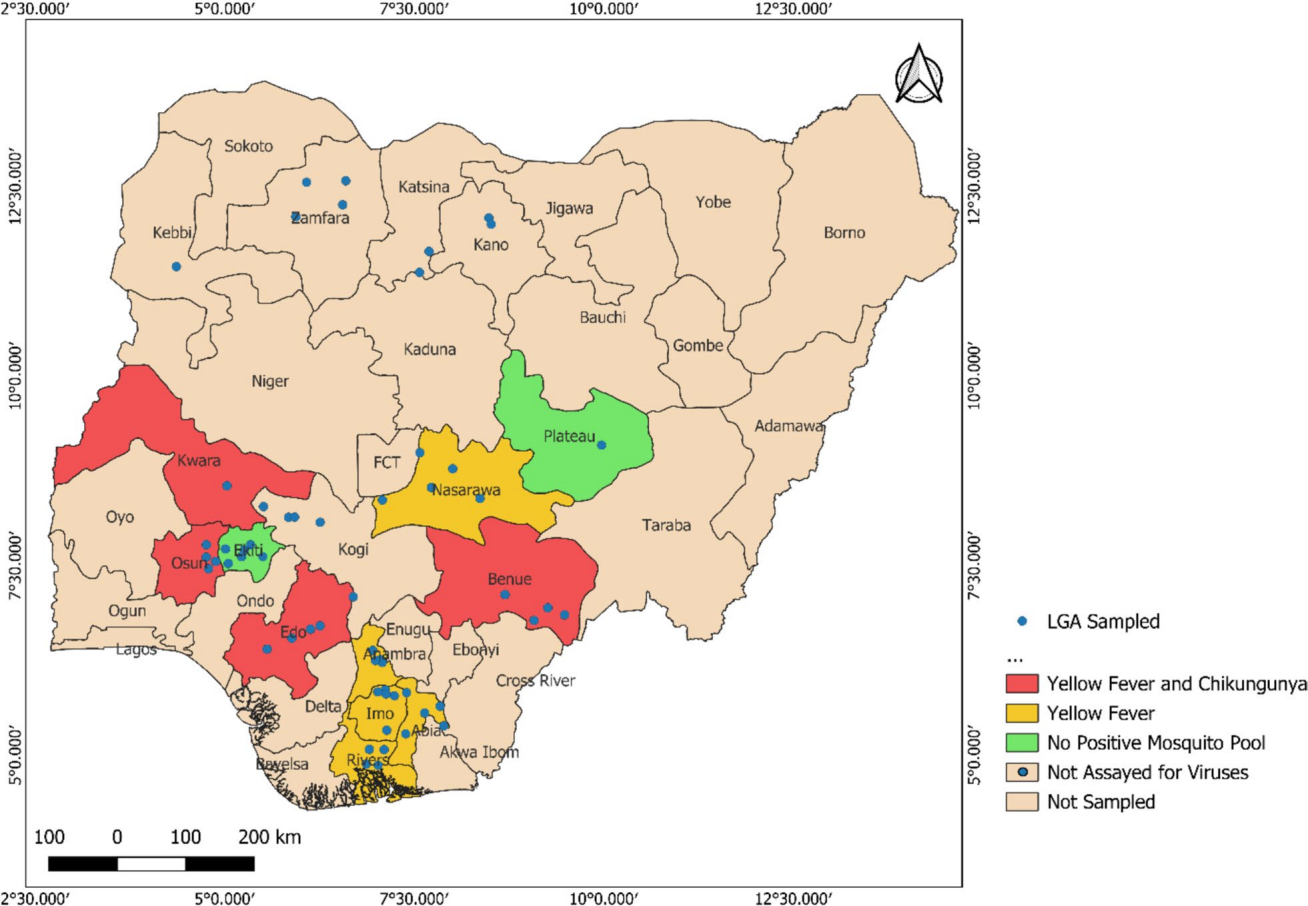
Map of Nigeria showing states where positive mosquito pools were detected

Of the six arboviruses screened, only YFV and CHIKV were detected from the 11 states (Fig. 5). All mosquito pools from all sites where vectors were assayed tested negative for dengue, Zika, o’nyong’nyong, and West Nile viruses.

**Fig. 6:**
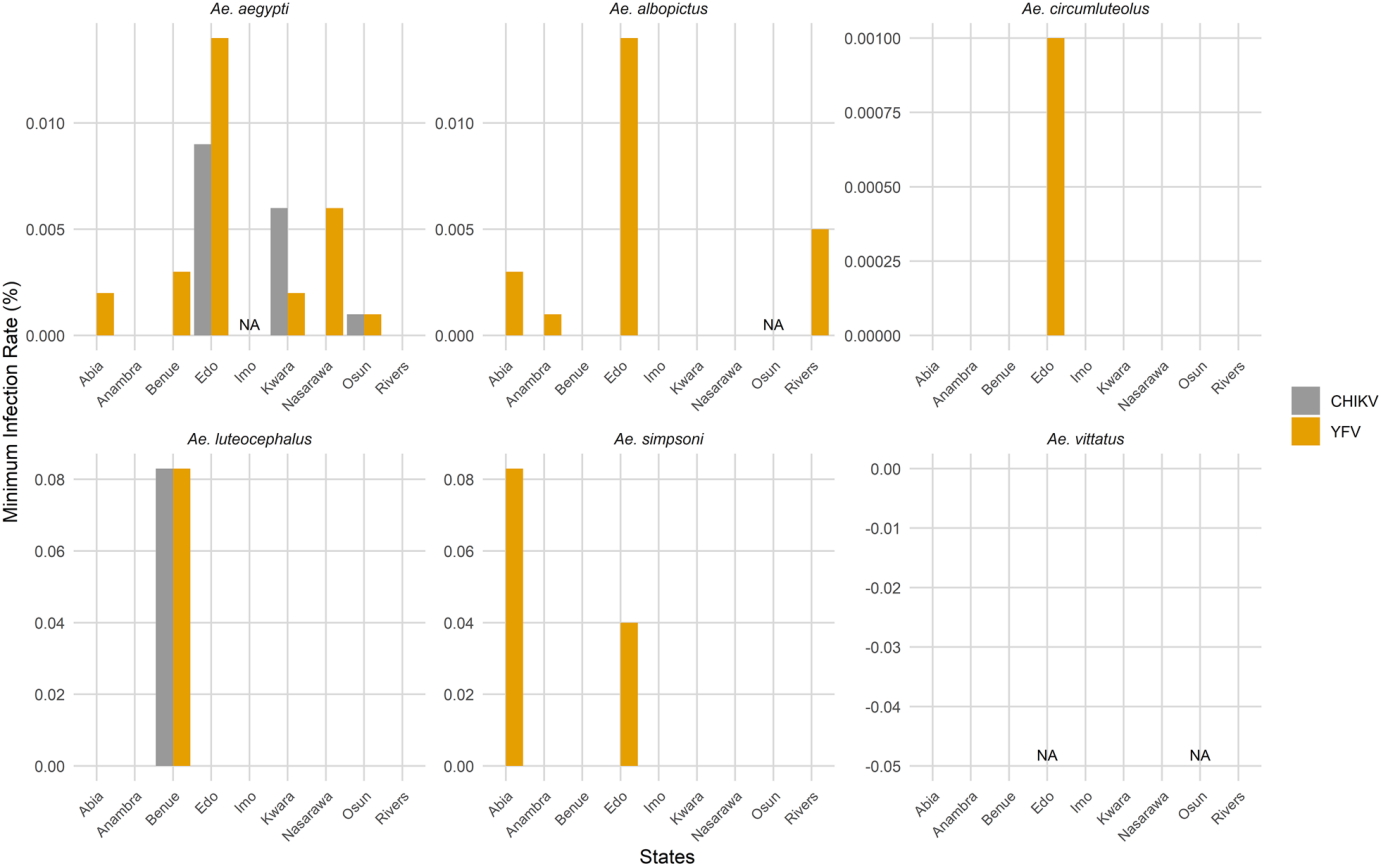
Minimum infection rates (MIR) of yellow fever and chikungunya viruses by state and *Aedes* species. “NA” indicates cases where MIR could not be estimated because the number of positive pools equaled the number of pools tested

The infection rates of yellow fever and chikungunya viruses in individual *Aedes* species for each state are presented in Fig. 6, while the overall minimum infection rates across all sampled states are summarized in Table 2. Nine (82%) of the 11 states where *Aedes* mosquito pools were assayed for viruses recorded positive pools for YFV (Table 2a). Edo State recorded the highest prevalence (64%) of YFV-positive mosquito pools. The infection rate per state ranged from 0.5 (Anambra) to 55.6 (Imo) per 1000 mosquitoes (Table 2a). Only 2 of the 11 states (Plateau and Ekiti states), accounting for 18% of the states, had no YFV-positive mosquitoes.

**Table 2:** Yellow fever and chikungunya minimum infection rates across the states

No.	State	Number of mosquitoes	Number of pools	No. of positive pools	Minimum infection rate	Lower	Upper
(a) Yellow fever infection rate across the states							
1	Abia	184	21	4	0.0034	7.410142 × 10^−5^	0.006757666
2	Anambra	938	6	1	0.0005	0.000000 × 10^0^	0.001580054
3	Imo	11	4	1	0.0556	0.000000 × 10^0^	0.161374590
4	Edo	670	22	14	0.0083	3.967394 × 10^−3^	0.012620284
5	Rivers	74	6	3	0.0082	0.000000 × 10^0^	0.017433923
6	Osun	194	14	3	0.0018	0.000000 × 10^0^	0.003782096
7	Benue	96	17	3	0.0036	0.000000 × 10^0^	0.007725023
8	Kwara	89	12	2	0.0019	0.000000 × 10^0^	0.004465553
9	Nasarawa	84	2	1	0.0060	0.000000 × 10^0^	0.017584060
(b) Chikungunya minimum infection rate across the states							
1	Benue	96	17	1	0.0012	0.000000000	0.003577725
2	Edo	670	22	2	0.0012	0.000000000	0.002825927
3	Kwara	89	12	6	0.0056	0.001135387	0.010100568
4	Osun	194	14	1	0.0006	0.000000000	0.001751115

On the other hand, only 4 (Kwara, Edo, Osun, and Benue) of the 11 states had mosquito pools positive for the chikungunya virus (Table 2b). The minimum infection rate per state ranged from 0.6 (Osun) to 5.6 (Kwara) per 1000 mosquitoes (Table 2b). The states are in the North Central (Benue and Kwara states, savannah/rainforest), South West (Osun, rainforest), and South South (Edo, rainforest) zones.

Of the seven *Aedes* mosquito species collected nationwide, only *Ae. africanus* recorded no YFV-positive pool. The minimum infection rate per species in each state is shown in Additional file 4: Table S1. However, the overall infection rate per species across the states ranged from 0.9 (*Ae. circumluteolus*) to 62.5 (*Ae. luteocephalus*) per 1000 mosquitoes (Table 3a). The infection risk associated with *Aedes aegypti* and *Ae. albopictus* (the most abundant species across the states) was observed to be 2.0 and 4.2 per 1000 mosquitoes, respectively. Regression analysis using the GLM showed a positive correlation between yellow fever infection and the number of pools, but not the number of mosquitoes in the pools (Additional file 5: Table S2). The southern part of Nigeria, where there were more and larger yellow fever outbreaks, accounted for more of these pools and a greater mosquito richness.

**Table 3:** Yellow fever and chikungunya minimum infection rates per species

No.	Mosquito species	Number of mosquitoes	Number of pools		No. of positive pools	Minimum infection rate		Lower	Upper
(a) Yellow fever minimum infection rate per species									
1	*Ae. aegypti*	1004	62		11	0.0020		0.0008155673	0.003166333
2	*Ae. albopictus*	765	32		10	0.0042		0.0015855957	0.006726956
3	*Ae. simpsoni* complex	22	8		4	0.0455		0.0019340610	0.088975030
4	*Ae. circumluteolus*	542	3		1	0.0009		0.0000000000	0.002734804
5	*Ae. luteocephalus*	10	3		1	0.0625		0.0000000000	0.181107935
6	*Ae. vittatus*	28	5		3	0.5556		0.0000000000	0.116650204
7	*Ae*. species	19	8		2	0.0213		0.0000000000	0.050448530
(b) Chikungunya minimum infection rate per species									
1	*Ae. aegypti*	1004		62	9		0.0016	0.0005655926	0.002692326
2	*Ae. luteocephalus*	10		3	1		0.0625	0.0000000000	0.181107935

CHIKV-positive pools were only detected in two *Aedes* species (*Ae. aegypti* and *Ae. luteocephalus*). *Ae. aegypti* accounted for 90% of all the CHIKV-positive pools across the states (Table 3b). However, the minimum infection rate of chikungunya virus for *Ae. aegypti* and *Ae. luteocephalus* was 1.6 and 62.5 per 1000 mosquitoes, respectively (Table 3b). Regression analysis showed no correlation between the infectivity of mosquitoes with CHIKV and the number of mosquitoes in the pools or the number of pools tested. This is also shown in Additional file 5: Table S2.

### Evidence of vertical transmission of yellow fever virus

Some adult mosquitoes that emerged from larval surveys were found to be YF-positive (Additional file 6: Table S3). These include *Aedes aegypti* from Kwara (Gaa Alasoro), Osun (Iloo-Ijesa), Edo (Irrua), Nasarawa (Majaga), and Imo (Okigwe) states. Also, *Ae. vittatus* and *Ae. simpsoni* complex, which emerged from larvae from Ivioghe and Irrua (both in Edo State), respectively, tested positive for YFV. There was no evidence of vertical transmission in CHIKV-positive mosquitoes.

## Discussion

This study investigated the presence of different arbovirus vectors (mosquitoes) and their possible roles in the transmission of arboviruses during yellow fever outbreaks in Nigeria between 2017 and 2020. Flaviviruses (yellow fever, West Nile, dengue, and Zika viruses) and alphaviruses (chikungunya and o’nyong’nyong viruses) were screened across sites and mosquito pools.

This study’s findings revealed the presence of several established vectors of yellow fever and other arboviruses across Nigeria’s different ecological and geographical zones. Though the study was cross-sectional, there was a decrease in the richness of *Aedes* species from the south to the north and some disparity according to individual state data recorded during previous studies in the same zone [14, 36, 47]. However, it is noteworthy that more states were sampled in the southern parts of the country, in response to yellow fever outbreaks. The rainforest South East (Abia, Anambra and Imo states) as well the South South (Edo and Rivers states) zones seem to be the richest ecozone in terms of *Aedes* mosquito diversity, accounting for all seven species (*Aedes aegypti*, *Ae. albopictus*, *Ae. africanus*, *Ae. luteocephalus*, *Ae. simpsoni* complex, *Ae. circumluteolus*, and *Ae. vittatus*) collected as adult mosquitoes during the study. Six species were collected in Edo State, which was found to have the greatest richness of all the states. On the other hand, all the adults collected from mHLC in Rivers State were *Aedes albopictus*. This is contrary to previous findings [48], where only *Ae. aegypti* was found from larval surveys. Notably, recent unpublished findings from our field teams in the states bordering Rivers State to the east (Akwa Ibom State) and west (Bayelsa State) indicate complete dominance of *Aedes albopictus* in both rural and urban areas of the rainforest/mangrove habitats.

It is noteworthy that *Ae. circumluteolus* was dominant in Agenebode Community of Edo State. The team discontinued collection by mHLC because of the unbearable number of *Aedes circumluteolus* alighting to bite collectors. Hence, only the CDC light trap was deployed thereafter. The unusual dominance of the biting population of *Ae. circumluteolus* in Agenebode is, to our knowledge, the first record of such an occurrence in Africa, as not so much information on this species is recorded, owing to its relatively small usual numbers during surveillance. A study that spanned 7 years across several states in Nigeria only recorded 40 *Aedes circumluteolus* from 7 of the 15 states where surveillance was carried out, providing a measure of how uncommon this species is in Nigeria [14]. On the contrary, another study [49] recorded large numbers of *Ae. circumluteolus* from vegetation in Tongaland, now part of modern-day Eswatini (formerly Swaziland). However, in stark contrast to our findings, their report indicated only very small numbers of mosquitoes biting humans. In addition to the main arbovirus vectors, the potential role of *Aedes circumluteolus* should not be overlooked. In other African countries, particularly South Africa, several arboviruses (Ndumu, Spondweni, Wesselsbron, and Bunyamwera) have been detected in this species, which is also regarded as a potential vector of Rift Valley fever [50]. Although the present study did not investigate these viruses, the notable abundance of *Ae. circumluteolus* in Edo State raises important questions about its possible contribution to arbovirus transmission in that part of Nigeria. This underscores the need for further entomological and virological studies focusing on *Ae. circumluteolus* to clarify its epidemiological importance.

The two South West states (Osun and Ekiti) sampled during this study reported three species (*Ae. aegypti*, *Ae. albopictus*, and *Ae. vittatus*). These data agree with previous findings [51] in Oshogbo Metropolis (Osun State), where the three *Aedes* species were also found. However, this contrasts with the work of Chukwuekezie and colleagues [14], who collected five *Aedes* species (including *Ae. aegypti* and *Ae. albopictus*) in a study carried out in the neighboring Ondo State in 2010. In addition to the three vectors that we recorded in the South West, [14] also reports collection of *Ae. luteocephalus* in a 2011 study in Ogun State.

Of the five *Aedes* species (*Ae. aegypti*, *Ae. albopictus*, *Ae. luteocephalus*, *Ae. vittatus*, and *Ae. africanus*) collected across the Guinea savannah–North Central (NC) zone, three species were present in each of Kwara, Nasarawa, and Plateau states, while Benue State recorded four species. This is in line with the findings of [14], which reported eight and six different *Aedes* species from Benue State in studies carried out in 2007 and 2010, respectively. Previous studies have also found up to five or more *Aedes* species in Benue State [16, 52]. The richness of very important arbovirus vectors almost always collected in studies in Benue State may be responsible for the seemingly regular outbreaks recorded in the state for decades.

Considering the very dry nature of the Sahel savannah–North West zone and the timing (skewed toward the dry season) of the responses, sampling in Kano and Kebbi states recorded no mosquitoes. Collections were only made in Katsina and Zamfara states, where only *Ae. aegypti* was collected. Despite the timing of the collection, it is important to note the prevailing climatic conditions in the North West zone. The area is characterized by high temperatures and low humidity, which reduce egg thermal tolerance. These conditions generally affect the survival and longevity of mosquito species such as *Aedes albopictus* more than *Ae. aegypti* [53–55]. Consequently, this may have significantly influenced the mosquito species richness in the area. This agrees with previous [14] and recent [47] reports of the dominance of *Ae. aegypti* in the northern states of Nigeria. There have been no reports of *Aedes albopictus* in the far north, a region that is predominantly inhabited by the seemingly better-adapted *Aedes aegypti*.

Larval sampling in this study was primarily conducted using domestic and peridomestic natural and artificial containers. Our findings revealed that the most infested containers were artificial, found in both domestic and peridomestic environments. The domestic containers were mainly plastic water storage containers and earthen pots, while the peridomestic containers consisted largely of used tyres, discarded containers, and plant axils. Similar findings have been reported in Garoua, Cameroon [56] and Enugu, Nigeria [57], where domestic and peridomestic containers served as the primary breeding sites. Additionally, our study identified used/discarded tyres containing immature stages of mosquitoes across several states. These tyres emerged as the preferred breeding sites for mosquitoes in most locations sampled, aligning with previous reports [56, 57] of large proportions of collections from used/discarded tyres. Notably, a significant proportion of *Aedes vittatus* was collected from these tyres, further supporting the predilection of this species for such sites, as observed in Ethiopia [58]. In our study, *Aedes luteocephalus*, an established tree-hole breeder, was unexpectedly found during domestic and peridomestic container collections in Benue State. Although rare, this occurrence mirrors a previous accidental finding in Enugu, Nigeria [57], where *Ae. luteocephalus* and *Ae. africanus* were found breeding in household water storage containers in Udi Hills, Enugu.

Findings from this study revealed that all the larval indices were higher than the standard threshold in all but one (Kogi) of the states where the indices were calculated. The high larval indices observed across the study sites agree with previous reports within [30] and outside [59] Nigeria. Most of the sites carried a high epidemic risk, as they were outbreak locations. Also, established arbovirus vectors were present in these areas, further elevating the risk of transmission.

*Aedes aegypti* is typically regarded as the urban vector for yellow fever and other arboviruses, owing to its adaptation to urban areas and overwhelming presence [13]. However, surveillance in the two urban areas covered in this study suggests otherwise. *Aedes albopictus* was the only human-biting *Aedes* species collected from various parts of Port Harcourt, Rivers State (South South zone) and Awka, Anambra State (South East zone). Our finding suggests a gradual intraspecific displacement of the once-dominant *Ae. aegypti* by *Ae. albopictus* in some urban areas of Nigeria. This trend aligns with the findings of Chukwuekezie and colleagues [14], who documented a steady increase in the density of *Ae. albopictus* in urban Enugu between 2008 and 2014, with the species becoming dominant by 2012–2014. The spread of *Ae. albopictus* appears to be extending beyond the city, into Enugu’s suburbs and rural areas. While reports from several countries in Africa and Asia have shown *Ae. aegypti* dominance in cities and *Ae. albopictus* dominance in suburban and rural areas [56, 60], there are exceptions. For instance, previous reports from the Republic of the Congo [61] and Cameroon [62, 63] noted a gradual replacement of native *Ae. aegypti* by the invasive *Ae. albopictus*. These findings support the results of this study and suggest that, with robust surveillance systems, similar trends could be uncovered in other parts of Africa.

In contrast, Mukhtar and Ibrahim [47] reported only *Ae. aegypti* in their collections from cities in Kano and Bauchi states, in northern Nigeria, a region characterized by higher temperatures and lower humidity. This may be attributed to the high mortality of *Ae. albopictus* eggs in these conditions [53]. *Aedes albopictus* is known to thrive in areas with annual mean temperatures between 5 °C and 28.5 °C [2, 54] and relative humidity of 52% and above [55].

The yellow fever vaccination coverage in Nigeria (54%) is insufficient to achieve herd immunity and prevent outbreaks [64]. This inadequate coverage is a key reason for the recurring outbreaks across the country, as infected mosquitoes are present in various states, and a large portion of the population remains unvaccinated. In this study, yellow fever virus (YFV) was detected in mosquito pools from 9 of the 11 states where samples were assayed. Overall, about 26% of the pools tested positive for the virus. Notably, YFV was found in mosquito pools from four of Nigeria’s six geographical zones (mostly rural areas), reflecting the country’s diverse ecological regions. The two zones not represented, located in the drier, northern parts of the country, had very few mosquito samples because the areas were too dry by the time surveillance and response efforts were carried out. Our findings align with reports that yellow fever outbreaks in Africa are largely rural or sylvatic in nature [13]. The predominance of cases in these settings underscores the role of sylvatic and peridomestic *Ae*. species in facilitating spillover and sustaining transmission, and it highlights the vulnerability of rural populations to epidemic spread.

*Aedes aegypti* and *Ae. albopictus* were the two most abundant species across the study sites. These species are likely the primary vectors responsible for the recent yellow fever transmission cycles in Nigeria, accounting for about 64% of all positive pools. Notably, while *Ae. aegypti* had the highest number of positive pools overall, only about 19% of its pools tested positive for viruses. In contrast, over 31% of *Ae. albopictus* pools tested positive for YFV. This correlates with the more than double minimum infection rate of *Ae. albopictus* versus *Ae. aegypti* observed in this study. This finding is particularly significant, as wild populations of *Ae. albopictus* infected with yellow fever had not been reported [62] until the year 2018, when the first-ever case was documented [63]. (http://www.iec.gov.br/portal/descoberta/). To our knowledge, this study provides the second documentation ever in the world of wild populations of *Ae. albopictus* found to be infected with YFV. This finding is significant because the invasive species [65] is known to be a potent transmitter of yellow fever in laboratory settings [62, 66]. Furthermore, our findings regarding other YFV-infected vectors align with previous reports from the 1969 yellow fever outbreak in Jos, Plateau State, where *Ae. luteocephalus* was primarily involved in virus transmission [15]. Similarly, more recent studies in Benue State identified *Ae. aegypti* [52] and *Ae. luteocephalus* as vectors of YFV [16]. The consistent detection of YFV in *Ae. luteocephalus* across the North Central zone of Nigeria suggests that this species may have been involved in yellow fever transmission in this region of Nigeria for over 40 years.

*Aedes aegypti*, which has a wider distribution in Nigeria [14], had positive pools in seven of the nine states, spanning both southern and northern regions. In contrast, *Ae. albopictus* recorded positive pools in five states, all located in the southern part of the country. *Ae. albopictus* appears to be more established in the southern regions, which are wetter and more forested. A recent study [47] in Kano and Bauchi states (North West and North East Nigeria) did not record any *Ae. albopictus*, reinforcing earlier reports that this species is not as well established in northern Nigeria. It is, therefore, unsurprising that no *Ae. albopictus* pools tested positive for yellow fever in the three North Central states where pools were assayed. Our findings suggest a correlation between vector density and yellow fever minimum infection rates, with higher rates of infected pools found in the wetter southern regions and the transition zone (North Central). This correlation is further supported by the larger and more frequent yellow fever outbreaks recorded in these areas [30, 31].

Our study is the first to report the presence of CHIKV in mosquitoes from yellow fever outbreak locations in Nigeria, suggesting the possibility of co-circulation of both diseases in these areas. CHIKV was detected in mosquito pools from 4 out of the 11 states where samples were assayed, with approximately 7% of the pools testing positive for the virus. The virus was found in mosquito pools from three of Nigeria’s six geographical zones. Although no pools were tested from the North East zone, circulation of CHIKV had previously been reported in febrile patients visiting hospitals in Borno State [25]. This suggests that, with proper surveillance and diagnostic measures, the virus may be detected in mosquitoes across the country. Similar to yellow fever, positive CHIKV pools were more frequent in the southern and North Central zones, which have higher vector densities and larger outbreaks. This highlights the relationship between minimum infection rates and vector density in relation to the ecogeographical characteristics of the country.

CHIKV was detected in *Aedes aegypti* and *Ae. luteocephalus* pools from four states. The virus was detected in *Aedes aegypti* pools from three of the four states (Edo, Kwara, and Osun states), while it was detected in *Ae. luteocephalus* only in Benue State. Both species have been frequently observed to be naturally infected with CHIKV in Senegal [67, 68]. Our finding is consistent with a previous report of CHIKV circulating among febrile patients in Kwara State [26]. Owing to the widespread distribution of *Ae. albopictus* in Nigeria, it has been suggested to be involved in CHIKV transmission in Nigeria [69]. However, our study did not detect CHIKV in any of the *Ae. albopictus* pools, which contrasts with previous assumptions [63]. The widespread presence of the two major arbovirus vectors in Nigeria, combined with the detection of CHIKV in *Ae. aegypti*, underscores the urgent need for proactive measures to prevent a potential outbreak and the spread of the disease in Nigeria.

It is evident that the arbovirus vectors responsible for globally important diseases abound in Nigeria, spanning from the drier northern regions to the wetter southern regions. These established vectors include two major and several secondary vectors of arboviruses. Owing to variations in climatic characteristics across these regions, there is a geographical skew favoring the breeding of these vectors in the southern and North Central parts of the country. Consequently, the risk of infection is higher in these regions, given the density and richness of mosquitoes, the timing of collection, as well as the number and size of positive pools recorded in our study. Ultimately, this geographical skew is reflected in the frequency and magnitude of outbreaks reported from these areas.

Our study had some limitations. Variations in trap deployment, seasonal differences in sampling, and the number of containers examined per site may have influenced mosquito richness, abundance, and breeding habitat assessment. The cross-sectional design and pooled trap processing further limited the temporal and spatial resolution of species distribution. Using whole mosquitoes for viral assays also prevented definitive conclusions on transmission potential. In addition, the relatively high number of positive pools without sequencing confirmation may have introduced uncertainty in virus identification. Future studies with standardized trap deployment, longitudinal sampling, individual trap processing, sequencing validation, assays targeting salivary glands, and consistent container sampling are needed to better characterize species dynamics and arbovirus transmission potential.

## Conclusions

The recurrent outbreaks of yellow fever in Nigeria, coupled with the detection of both yellow fever virus (YFV) and chikungunya virus (CHIKV) in *Aedes* mosquito pools (including instances of co-detection within the same pools), underscore the endemic co-circulation of these arboviruses. This study identified several critical risk factors contributing to YFV transmission. Specifically, *Aedes aegypti* and *Aedes albopictus* were the primary vector species associated with virus-positive pools. High-density, productive larval habitats, particularly tyres, discarded materials, and water storage containers, were significant contributors to YFV risk. Furthermore, the geographical distribution of outbreaks and infected mosquito pools was notably higher in the wetter southern regions of Nigeria. These findings highlight the complex interplay of environmental, ecological, and vector-related factors in the persistence and spread of YFV. The observed co-detection of YFV and CHIKV in mosquito pools suggests potential implications for mixed virus transmission dynamics. Given the low vaccination coverage and the widespread distribution of high-risk vector habitats, it is imperative to enhance surveillance and control measures targeting these key risk factors to mitigate future outbreaks.

## Supplementary Information

Supplementary material 1. Container preferences of *Aedes* species across Nigeria

Supplementary material 2. Container preferences of *Aedes* species in northern Nigeria

Supplementary material 3. Container preferences of *Aedes* species in southern Nigeria

Supplementary material 4. Minimum infection rates by state and species

Supplementary material 5. Regression analysis

Supplementary material 6. Evidence of vertical transmission

Supplementary material 7. *Aedes* species by location and collection methods

## Data Availability

All data supporting the conclusions of this article are provided within the text and supplementary material.

## Abbreviations

AFI: Acute febrile illness
BG-Sentinel trap: Biogents sentinel trap
BI: Breteau Index
CDC: Centers for Disease Control and Prevention
CHIKV: Chikungunya virus
CI: Container index
Ct: Cycle threshold
DENV: Dengue virus
DMEM: Dulbecco’s modified Eagle’s medium
FCT: Federal Capital Territory
HI: House index
LGAs: Local Government Areas
mHLC: Modified human landing catch
NAVRC: National Arbovirus and Vectors Research Centre
NCDC: Nigeria Centre for Disease Control
NTC: Nontemplate control
ONNV: O’nyong’nyong virus
RT-qPCR: Reverse-transcription quantitative polymerase chain reaction
SPSS: Statistical Package for the Social Sciences
UV: Ultraviolet
WHO: World Health Organization
WNV: West Nile virus
YF: Yellow fever
YFV: Yellow fever virus
ZIKV: Zika virus
