# Supplementary figures and images for: Nationwide surveillance detects yellow fever and chikungunya viruses in multiple Aedes mosquito species in Nigeria
Source: Parasit Vectors. 2025 Oct 31;18:443. doi: 10.1186/s13071-025-07051-z (PMC12577304; doi:10.1186/s13071-025-07051-z)

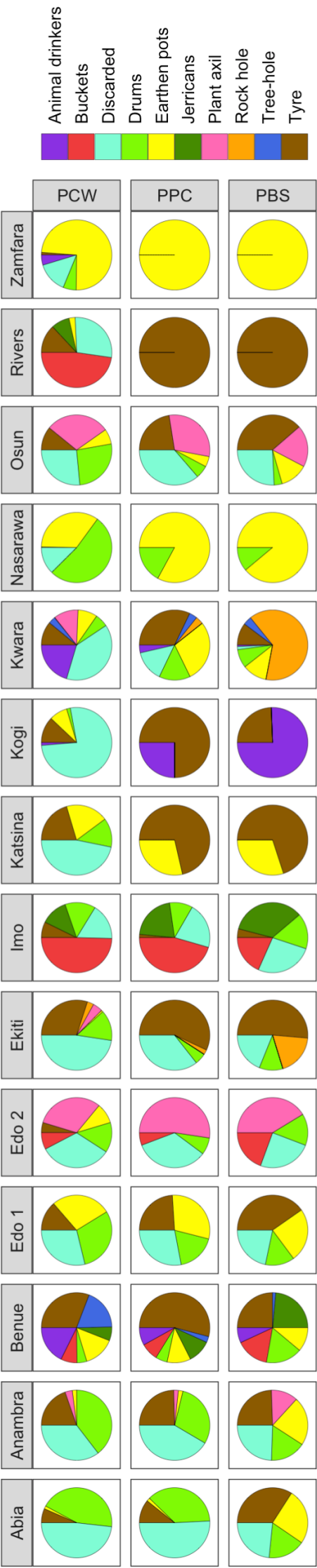

Supplement: Supplementary file 1 — Supplementary material 1. Container preferences of Aedes species across Nigeria [file 13071_2025_7051_MOESM1_ESM.pdf]

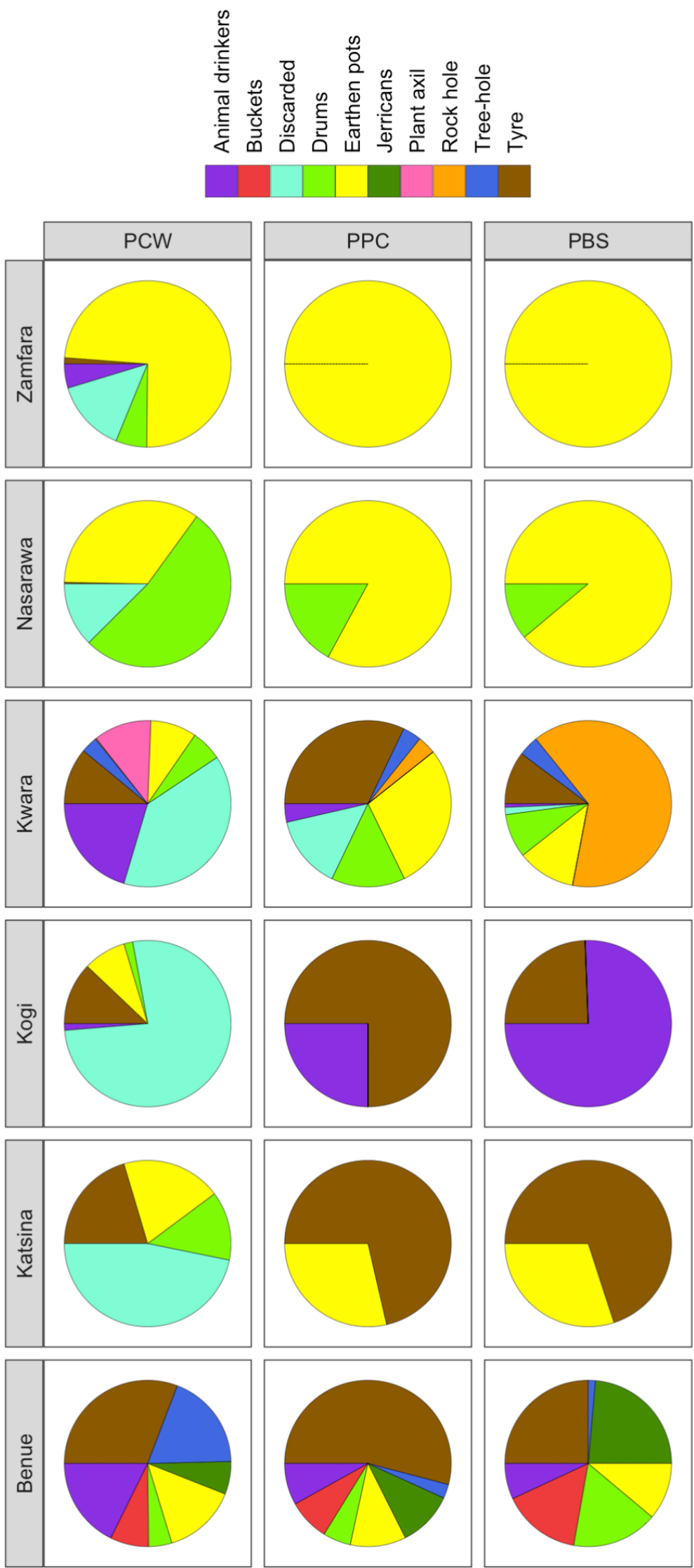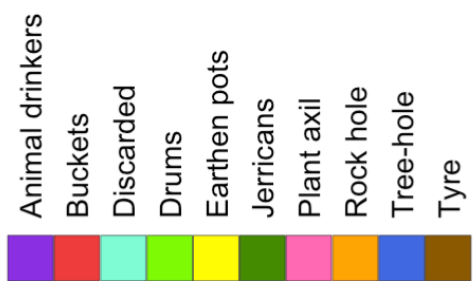

Supplement: Supplementary file 2 — Supplementary material 2. Container preferences of Aedes species in northern Nigeria [file 13071_2025_7051_MOESM2_ESM.pdf]

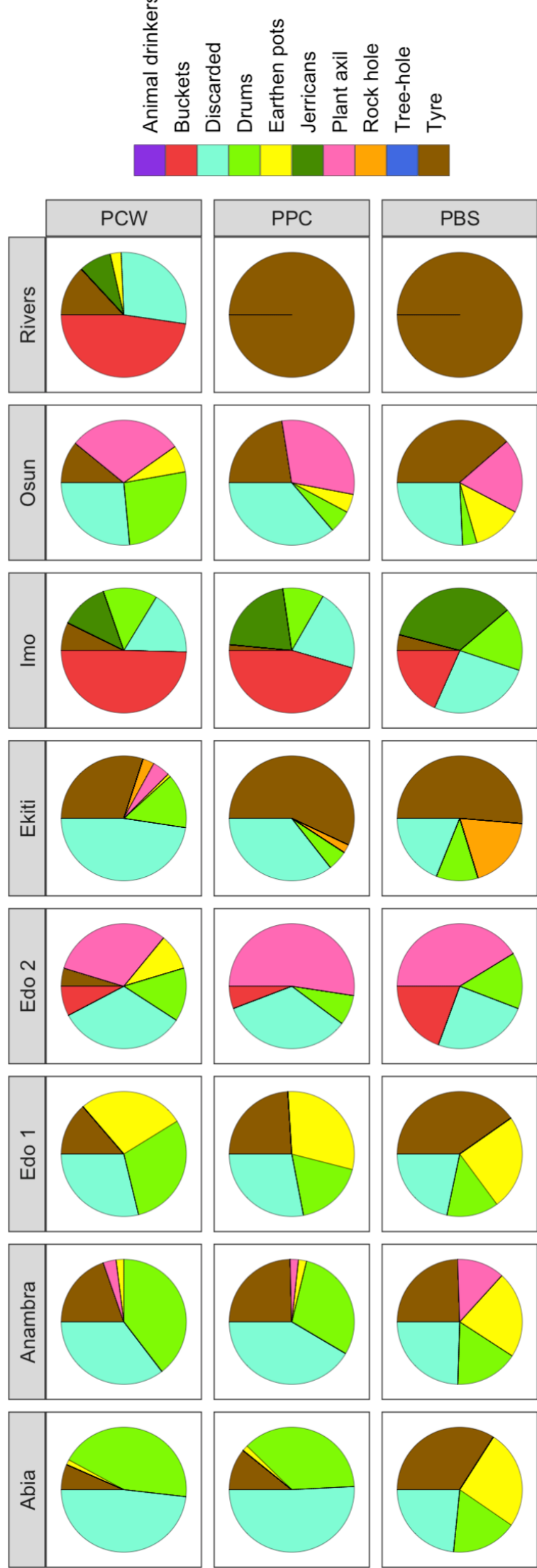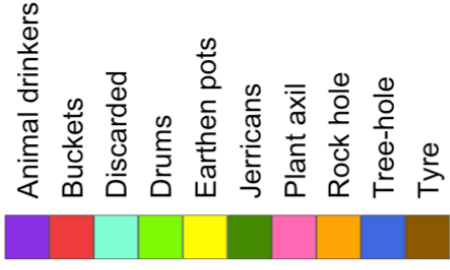

Supplement: Supplementary file 3 — Supplementary material 3. Container preferences of Aedes species in southern Nigeria [file 13071_2025_7051_MOESM3_ESM.pdf]
