# Supplementary material for: Nationwide surveillance detects yellow fever and chikungunya viruses in multiple Aedes mosquito species in Nigeria
Source: Parasit Vectors. 2025 Oct 31;18:443. doi: 10.1186/s13071-025-07051-z (PMC12577304; doi:10.1186/s13071-025-07051-z)
Supplement: Supplementary file 4 — Supplementary material 4. Minimum infection rates by state and species [file 13071_2025_7051_MOESM4_ESM.pdf]

Breeding sites vs risk of infection
YFV

Pearson Correlation coefficient with TyreContainer is 0.06093

==

	coef	std err	z	P>|z|	[0.025	0.975]
IR_YFV	0.0348	0.008	4.616	0.000	0.020	0.050
const	4.3987	0.066	66.983	0.000	4.270	4.527

Pearson Correlation coefficient with MetalContainer is 0.448

	coef	std err	z	P>|z|	[0.025	0.975]
IR_YFV	0.0175	0.010	1.728	0.084	-0.002	0.037
const	3.4957	0.095	36.699	0.000	3.309	3.682

Pearson Correlation coefficient with Plastic Containers is 0.309

==

	coef	std err	z	P>|z|	[0.025	0.975]
IR_YFV	0.0223	0.005	4.910	0.000	0.013	0.031
const	5.1884	0.042	124.290	0.000	5.107	5.270

YFV risk infection is positively correlated with Plastic Containers, Metal Container and Tyre Container

Earthenware:0.185

	coef	std err	z	P>|z|	[0.025	0.975]
IR_YFV	-0.1310	0.005	-25.041	0.000	-0.141	-0.121
const	2.6226	0.087	30.022	0.000	2.451	2.794

==

YFV risk infection is negatively correlated with Earthenware

Drinkers:nan

Leaf Axils:-0.142

	coef	std err	z	P>|z|	[0.025	0.975]
IR_YFV	-0.0144	0.013	-1.132	0.257	-0.039	0.011
const	2.4913	0.138	18.091	0.000	2.221	2.761

Tree holes:nan

Rockpool:nan

CHIKV

Tyre Container:0.358

	**coef**	**std err**	**z**	**P>|z|**	**[0.025**	**0.975]**
**IR_CHIVK**	**0.0618**	**0.006**	**11.202**	**0.000**	**0.051**	**0.073**
**const**	**4.9046**	**0.071**	**68.953**	**0.000**	**4.765**	**5.044**

Metal Container:-0.2732

IR_CHIVK	-0.0293	0.008	-3.630	0.000	-0.045	-0.013
const	2.9221	0.138	21.180	0.000	2.652	3.193

PlasticContainers:-0.139

	coef	std err	z	P>|z|	[0.025	0.975]
IR_CHIVK	-0.0135	0.003	-3.929	0.000	-0.020	-0.007
const	4.8204	0.056	85.558	0.000	4.710	4.931

Earthenware:-0.354

	coef	std err	z	P>|z|	[0.025	0.975]
IR_CHIVK	-0.1434	0.009	-15.679	0.000	-0.161	-0.125
const	1.6428	0.181	9.063	0.000	1.287	1.998

Drinkers:nan

LeafAxils:=0.259

	coef	std err	z	P>|z|	[0.025	0.975]
IR_CHIVK	0.0535	0.012	4.619	0.000	0.031	0.076
const	3.2917	0.154	21.343	0.000	2.989	3.594

Treeholes:nan

Rockpool:nan
