# Supplementary material for: Nationwide surveillance detects yellow fever and chikungunya viruses in multiple Aedes mosquito species in Nigeria
Source: Parasit Vectors. 2025 Oct 31;18:443. doi: 10.1186/s13071-025-07051-z (PMC12577304; doi:10.1186/s13071-025-07051-z)
Supplement: Supplementary file 6 — Supplementary material 6. Evidence of vertical transmission [file 13071_2025_7051_MOESM6_ESM.pdf]

Correlation between pool size and positivity Using regression

using regression on overall p value=0.427 for YFV and 0.472 CHIKV

YFV positivity vs number of pool

	coef	std err	z	P>|z|	[0.025	0.975]
Pos	0.2570	0.078	3.315	0.001	0.105	0.409
const	1.0430	0.136	7.661	0.000	0.776	1.310

YFV positivity is correlated with number of pools

YFV Positivity vs PoolSIze

	coef	std err	z	P>|z|	[0.025	0.975]
Pos	0.0156	0.020	0.794	0.427	-0.023	0.054
const	4.2980	0.029	150.230	0.000	4.242	4.354

No correlation between YFV positivity and Pool size

Mosquito pool size across species for YFV and CHIKV

	Coef.	Std.Err.	t	P>|t|	[0.025	0.975]
**Ae. aegypti**	42.883418	32.180316	1.332598	0.188061	-21.581502	107.348337
**Ae. africanus**	-47.480219	86.595720	-0.548298	0.585667	-220.952292	125.991854
**Ae. albopictus**	47.144781	35.748349	1.318796	0.192605	-24.467768	118.757330
**Ae. circumluteolus**	169.519781	56.860148	2.981346	0.004242	55.615218	283.424344
**Ae. luteocephalus**	-43.813552	71.616771	-0.611778	0.543161	-187.279184	99.652080
**Ae. simpsoni**	-41.146886	52.527184	-0.783345	0.436725	-146.371479	64.077708
**Ae. spp**	-39.480219	62.801540	-0.628650	0.532134	-165.286822	86.326384
**Ae. vittatus**	-39.146886	52.527184	-0.745269	0.459226	-144.371479	66.077708
**const**	48.480219	19.729372	2.457261	0.017123	8.957537	88.002901

Aedes circumluteolus had significantly more pool size (p-value =0.004242)

Mosquito pool size across species for YFV

	Coef.	Std.Err.	t	P>|t|	[0.025	0.975]
**Ae. aegypti**	46.883418	50.769229	0.923461	0.364959	-57.899122	151.665957
**Ae. africanus**	-43.480219	134.944783	-0.322207	0.750085	-321.992562	235.032124
**Ae. albopictus**	51.144781	56.247101	0.909287	0.372240	-64.943530	167.233092
**Ae. circumluteolus**	138.186448	82.135270	1.682425	0.105454	-31.332418	307.705313
**Ae. luteocephalus**	-40.480219	134.944783	-0.299976	0.766778	-318.992562	238.032124
**Ae. simpsoni**	-37.146886	82.135270	-0.452265	0.655140	-206.665751	132.371980
**Ae. spp**	-35.480219	98.041703	-0.361889	0.720602	-237.828348	166.867911
**Ae. vittatus**	-35.146886	82.135270	-0.427915	0.672529	-204.665751	134.371980
**const**	44.480219	31.847396	1.396667	0.175297	-21.249577	110.210015

No significant differences in pool size among species

Mosquito pool size across species for CHIKV2 as a reference

	Coef.	Std.Err.	t	P>|t|	[0.025	0.975]
Ae. aegypti	-126.636364	66.987606	-1.890445	0.063877	-260.828664	7.555937
Ae. africanus	-217.000000	113.124830	-1.918235	0.060185	-443.616266	9.616266
Ae. albopictus	-122.375000	69.274528	-1.766522	0.082758	-261.148555	16.398555
Ae. luteocephalus	-213.333333	98.743447	-2.160481	0.035029	-411.140227	-15.526440
Ae. simpsoni	-210.666667	81.873741	-2.573068	0.012757	-374.679478	-46.653855
Ae. spp	-209.000000	90.701648	-2.304258	0.024941	-390.697234	-27.302766
Ae. vittatus	-208.666667	81.873741	-2.548640	0.013583	-372.679478	-44.653855
const	218.000000	60.467765	3.605227	0.000664	96.868511	339.131489

Mosquito pool size across State for YFV and CHIKV

	Coef.	Std.Err.	t	P>|t|	[0.025	0.975]
Abia	-12.740079	46.719163	-0.272695	0.786147	-106.446853	80.966695
Anambra	175.759921	46.719163	3.762052	0.000423	82.053147	269.466695
Benue	-26.740079	52.895485	-0.505527	0.615285	-132.834986	79.354828
Edo	36.974206	37.304715	0.991140	0.326121	-37.849575	111.797988
Ekiti	-42.406746	52.895485	-0.801708	0.426302	-148.501653	63.688161
Imo	-55.073413	52.895485	-1.041174	0.302521	-161.168320	51.021494
Kwara	30.259921	87.863243	0.344398	0.731910	-145.971426	206.491267
Nasarawa	25.259921	87.863243	0.287491	0.774857	-150.971426	201.491267
Osun	5.926587	52.895485	0.112043	0.911212	-100.168320	112.021494
Plateau	-56.740079	87.863243	-0.645777	0.521210	-232.971426	119.491267
Rivers	-21.740079	63.469966	-0.342525	0.733310	-149.044709	105.564551
const	58.740079	18.355484	3.200138	0.002321	21.923641	95.556518

Anambra is the state with higher pool size (pvalue=0.000423).

Mosquito pool size across State for YFV and CHIKV 2 Anambra as reference

	Coef.	Std.Err.	t	P>|t|	[0.025	0.975]
Abia	-188.500000	66.556858	-2.832165	0.006523	-321.996152	-55.003848
Benue	-202.500000	71.889623	-2.816818	0.006799	-346.692324	-58.307676
Edo	-138.785714	58.996349	-2.352446	0.022396	-257.117405	-20.454023
Ekiti	-218.166667	71.889623	-3.034745	0.003726	-362.358990	-73.974343
Imo	-230.833333	71.889623	-3.210941	0.002250	-375.025657	-86.641010
Kwara	-145.500000	105.235633	-1.382612	0.172582	-356.575949	65.575949
Nasarawa	-150.500000	105.235633	-1.430124	0.158551	-361.575949	60.575949
Osun	-169.833333	71.889623	-2.362418	0.021860	-314.025657	-25.641010
Plateau	-232.500000	105.235633	-2.209328	0.031495	-443.575949	-21.424051
Rivers	-197.500000	81.515171	-2.422862	0.018848	-360.998727	-34.001273
const	234.500000	47.062806	4.982703	0.000007	140.103966	328.896034

Mosquito pool size across State for YFV 2 Anambra as reference

	Coef.	Std.Err.	t	P>|t|	[0.025	0.975]
**Abia**	-188.500000	105.735567	-1.782749	0.089091	-408.389150	31.389150
**Benue**	-202.500000	114.207466	-1.773089	0.090720	-440.007427	35.007427
**Edo**	-138.785714	93.724563	-1.480783	0.153513	-333.696613	56.125185
**Ekiti**	-218.166667	114.207466	-1.910266	0.069851	-455.674094	19.340760
**Imo**	-230.833333	114.207466	-2.021176	0.056192	-468.340760	6.674094
**Kwara**	-145.500000	167.182611	-0.870306	0.393973	-493.175273	202.175273
**Nasarawa**	-150.500000	167.182611	-0.900213	0.378215	-498.175273	197.175273
**Osun**	-169.833333	114.207466	-1.487060	0.151860	-407.340760	67.674094
**Plateau**	-232.500000	167.182611	-1.390695	0.178881	-580.175273	115.175273
**Rivers**	-197.500000	129.499094	-1.525107	0.142152	-466.808108	71.808108
**const**	234.500000	74.766337	3.136438	0.004986	79.014891	389.985109

Number of pools across State for YFV kwara as reference

	Coef.	Std.Err.	t	P>|t|	[0.025	0.975]
**Abia**	-6.750000	3.694219	-1.827179	0.081920	-14.432548	0.932548
**Anambra**	-10.500000	3.694219	-2.842279	0.009757	-18.182548	-2.817452
**Benue**	-6.333333	3.815372	-1.659952	0.111785	-14.267835	1.601168
**Edo**	-8.857143	3.532349	-2.507437	0.020447	-16.203064	-1.511222
**Ekiti**	-6.333333	3.815372	-1.659952	0.111785	-14.267835	1.601168
**Imo**	-10.666667	3.815372	-2.795708	0.010833	-18.601168	-2.732165
**Nasarawa**	-10.000000	4.672858	-2.140018	0.044251	-19.717740	-0.282260
**Osun**	-7.333333	3.815372	-1.922049	0.068272	-15.267835	0.601168
**Plateau**	-11.000000	4.672858	-2.354020	0.028386	-20.717740	-1.282260
**Rivers**	-9.000000	4.046814	-2.223972	0.037252	-17.415810	-0.584190
**const**	12.000000	3.304210	3.631731	0.001562	5.128520	18.871480

Number of pools across Specie for YFV, *Ae. aegypti* as reference group

	Coef.	Std.Err.	t	P>|t|	[0.025	0.975]
**Ae. africanus**	-4.727273	3.659867	-1.291651	0.208779	-12.280867	2.826322
**Ae. albopictus**	-1.727273	1.628195	-1.060851	0.299317	-5.087703	1.633157
**Ae. circumluteolus**	-4.060606	2.282328	-1.779151	0.087885	-8.771100	0.649888
**Ae. luteocephalus**	-4.727273	3.659867	-1.291651	0.208779	-12.280867	2.826322
**Ae. simpsoni**	-3.060606	2.282328	-1.341002	0.192474	-7.771100	1.649888
**Ae. spp**	-2.227273	2.693589	-0.826879	0.416451	-7.786568	3.332022
**Ae. vittatus**	-4.060606	2.282328	-1.779151	0.087885	-8.771100	0.649888
**const**	5.727273	1.056513	5.420922	0.000014	3.546738	7.907808

Mosquitos pool size across State for CHIKV, *Ae. aegypti* as reference

	Coef.	Std.Err.	t	P>|t|	[0.025	0.975]
**Ae. africanus**	-4.727273	3.661937	-1.290922	0.209028	-12.285138	2.830593
**Ae. albopictus**	-1.727273	1.629116	-1.060252	0.299584	-5.089603	1.635057
**Ae. circumluteolus**	-4.227273	2.695112	-1.568496	0.129857	-9.789711	1.335166
**Ae. luteocephalus**	-4.227273	2.695112	-1.568496	0.129857	-9.789711	1.335166
**Ae. simpsoni**	-3.060606	2.283619	-1.340244	0.192717	-7.773763	1.652551
**Ae. spp**	-2.227273	2.695112	-0.826412	0.416711	-7.789711	3.335166
**Ae. vittatus**	-4.060606	2.283619	-1.778145	0.088054	-8.773763	0.652551
**const**	5.727273	1.057110	5.417859	0.000014	3.545505	7.909041

Number of pools across State for CHIKV Kwara as reference

	Coef.	Std.Err.	t	P>|t|	[0.025	0.975]
**Abia**	-6.750000	3.694219	-1.827179	0.081920	-14.432548	0.932548
**Anambra**	-10.500000	3.694219	-2.842279	0.009757	-18.182548	-2.817452
**Benue**	-6.333333	3.815372	-1.659952	0.111785	-14.267835	1.601168
**Edo**	-8.857143	3.532349	-2.507437	0.020447	-16.203064	-1.511222
**Ekiti**	-6.333333	3.815372	-1.659952	0.111785	-14.267835	1.601168
**Imo**	-10.666667	3.815372	-2.795708	0.010833	-18.601168	-2.732165
**Nasarawa**	-10.000000	4.672858	-2.140018	0.044251	-19.717740	-0.282260
**Osun**	-7.333333	3.815372	-1.922049	0.068272	-15.267835	0.601168
**Plateau**	-11.000000	4.672858	-2.354020	0.028386	-20.717740	-1.282260
**Rivers**	-9.000000	4.046814	-2.223972	0.037252	-17.415810	-0.584190
**const**	12.000000	3.304210	3.631731	0.001562	5.128520	18.871480

Number of pools across Species for CHIKV as reference Aegypti

	Coef.	Std.Err.	t	P>|t|	[0.025	0.975]
**Ae. africanus**	-4.727273	3.661937	-1.290922	0.209028	-12.285138	2.830593
**Ae. albopictus**	-1.727273	1.629116	-1.060252	0.299584	-5.089603	1.635057
**Ae. circumluteolus**	-4.227273	2.695112	-1.568496	0.129857	-9.789711	1.335166
**Ae. luteocephalus**	-4.227273	2.695112	-1.568496	0.129857	-9.789711	1.335166
**Ae. simpsoni**	-3.060606	2.283619	-1.340244	0.192717	-7.773763	1.652551
**Ae. spp**	-2.227273	2.695112	-0.826412	0.416711	-7.789711	3.335166
**Ae. vittatus**	-4.060606	2.283619	-1.778145	0.088054	-8.773763	0.652551
**const**	5.727273	1.057110	5.417859	0.000014	3.545505	7.909041

==

	coef	std err	t	P>|t|	[0.025	0.975]
PoolSize	0.0002	0.003	0.077	0.939	-0.006	0.007
const	3.7943	0.499	7.607	0.000	2.797	4.791

No correlation between pool Size and number of pool

Species abundance and composition

YFV infection risk

Ae. aegypti:0.257

	coef	std err	z	P>|z|	[0.025	0.975]
YFV_aegypti	0.0754	0.004	18.067	0.000	0.067	0.084
const	4.8478	0.064	76.042	0.000	4.723	4.973

Ae. albopictus:0.5968

==

	coef	std err	z	P>|z|	[0.025	0.975]
YFV_albopictus	0.2060	0.006	34.374	0.000	0.194	0.218
const	6.1443	0.060	102.662	0.000	6.027	6.262

Ae. africanus:nan

	coef	std err	z	P>|z|	[0.025	0.975]
YFV_africanus	0.1021	0.028	3.672	0.000	0.048	0.157
const	-0.0049	0.001	-3.672	0.000	-0.008	-0.002

coef   std err   t   P>|t| [0.025   0.975]

***Ae. luteocephalus*:0.811**

	coef	std err	z	P>|z|	[0.025	0.975]
YFV_luteocephalus	0.1740	0.032	5.505	0.000	0.112	0.236
const	2.2205	0.467	4.759	0.000	1.306	3.135

***Ae. simpsopni*:0.8951**

	coef	std err	z	P>|z|	[0.025	0.975]
YFV_simpsopni	0.2984	0.053	5.659	0.000	0.195	0.402
const	3.1972	0.282	11.333	0.000	2.644	3.750

***Ae. simpsoni*: Infection rate is significantly correlated with *Ae. simpsoni* abundance**

***Ae. circumluteolus*:0.999**

	coef	std err	z	P>|z|	[0.025	0.975]
YFV_circumluteolus	0.6084	0.042	14.459	0.000	0.526	0.691
const	10.5277	0.299	35.178	0.000	9.941	11.114

***Ae. vittatus*:nan**

YFV is associated with species abundance

CHIKV infection risk

***Ae. aegypti*: 0.20**

	coef	std err	z	P>|z|	[0.025	0.975]
CHIKV_aegypti	0.0655	0.005	14.053	0.000	0.056	0.075
const	4.8650	0.084	58.156	0.000	4.701	5.029

***Ae. albopictus*:nan**

	coef	std err	z	P>|z|	[0.025	0.975]
CHIKV_albopictus	-0.1647	0.002	-94.620	0.000	-0.168	-0.161
const	0.0079	8.4e-05	94.620	0.000	0.008	0.008

***Ae. africanus*:nan**

Ae. luteocephalus:0.81

	coef	std err	z	P>|z|	[0.025	0.975]
CHIKV_luteocephalus	0.1740	0.032	5.505	0.000	0.112	0.236
const	2.2205	0.467	4.759	0.000	1.306	3.135

Ae. luteocephalus: infection rate is significantly correlated with the abundance

Ae. simpsopni:nan

	coef	std err	z	P>|z|	[0.025	0.975]
CHIKV_simpsopni	0.0040	0.010	0.400	0.689	-0.016	0.024
const	-0.0002	0.000	-0.400	0.689	-0.001	0.001

Ae. circumluteolus:nan

	coef	std err	z	P>|z|	[0.025	0.975]
CHIKV_circumluteolus	-0.1482	0.002	-71.730	0.000	-0.152	-0.144
const	0.0072	9.97e-05	71.730	0.000	0.007	0.007

Ae. vittatus:nan

	coef	std err	z	P>|z|	[0.025	0.975]
CHIKV_vittatus	-0.0071	0.009	-0.799	0.424	-0.025	0.010
const	0.0003	0.000	0.799	0.424	-0.001	0.001
